# Digital transformation in education: psychological perspectives on learning, engagement, and outcomes: a systematic review and meta-analysis

**DOI:** 10.3389/fpsyg.2026.1856031

**Published:** 2026-09-16

**Authors:** Pingping Hou

**Affiliations:** Huanghe University of Science and Technology, Zhengzhou, Henan, China

**Keywords:** cognitive psychology, digital transformation, educational technology, institutional factors, learning engagement, LMS, meta-analysis, motivation

## Abstract

**Background:**

The process of digital transformation has developed new educational systems that use technology-based tools, including learning management systems, artificial intelligence, and gamified systems, as well as digital teaching methods. The studies show incomplete evidence about how this phenomenon affects learning engagement, motivation, cognitive processes, self-regulation skills, and institutional performance.

**Objective:**

This meta-analysis aimed to synthesize global evidence on the psychological perspectives of digital transformation in education, focusing on learning engagement, motivation, cognitive/metacognitive processes, self-regulation, and institutional factors.

**Methodology:**

A systematic meta-analysis was conducted to assess 42 studies that employed a random-effects model with the inverse variance method as their analysis approach. The odds ratios to calculate effect sizes, which included 95% confidence intervals. The subgroup analyses were about five different psychological and institutional domains. The assessment of heterogeneity used I^2^ statistics, while the publication bias assessment relied on funnel plots and Egger’s regression test.

**Results:**

The results showed that digital transformation produced positive results in all areas which included learning engagement (OR = 1.79, 95% CI: 1.68–1.91), motivation, affective psychology (OR = 1.57, 95% CI: 1.21–2.03), cognitive and metacognitive psychology (OR = 1.79, 95% CI: 1.60–2.02) and self-regulation and learning psychology (OR = 1.66, 95% CI: 1.49–1.85) and institutional factors (OR = 1.71, 95% CI: 1.48–1.97). High motivation and self-regulation showed high domain heterogeneity, while the other domains maintained their consistency. The study showed no publication bias except for cognitive and metacognitive studies, which showed asymmetrical findings.

**Conclusion:**

Digital transformation in education brings about significant advantages for psychological and institutional results, which include better learning engagement and cognitive growth. The different factors that affect motivation and self-regulation show how contextual and pedagogical elements create an impact.

## Introduction

Educational systems today experience their most substantial change through digital transformation, which modifies all aspects of knowledge delivery and accessibility and educational experiences. Educational institutions now utilize digital technology through Learning Management Systems (LMS), artificial intelligence (AI), gamification tools, online platforms, and data-driven analytics to create new educational systems (Gao et al., 2026; Zhu et al., 2026; Huang et al., 2025; Sun et al., 2025). The recent developments have created more educational opportunities while establishing fresh methods for students to interact with others and learn in a customized way. Educational institutions need to create digital systems that maximize learning results because digital systems have become a standard practice in educational environments (Niță and Guțu, 2023; Prayogi et al., 2025). In the present analysis, “digital transformation” means the regular usage of digital technologies, strategies, applications or practices in accordance with teaching, learning, assessment, communication, engagement and educational management practices. The defined notion incorporates certain forms of technologies, such as LMS, online learning, blended learning, virtual education, multimedia learning, gamification, mLearning, assistive learning technologies and learning analytics but acknowledges that these forms are just various components of educational digital transformation.

The effects of digital transformation in psychology and education are multi-faceted. One of the most researched effects of student learning is learning engagement, which means that students can be engaged behaviorally, emotionally, and cognitively in the process of learning in school. Digital platforms can increase engagement by providing interactive content, instant feedback, collaboration capabilities, and individualized learning paths (Wang et al., 2026; Qiao et al., 2026; Olawumi and Onasanya, 2025). Nonetheless, the outcomes are not entirely beneficial. Inefficiently constructed digital settings may lead to distractions, overload of the brain, fatigue from screens, and technostress which might result in diminished attention spans (Enachescu, 2025). Digital environments can also affect students’ motivation and emotional experiences. Various aspects like gamification, autonomy, personalized feedback, and collaborative learning techniques can be beneficial for motivation, whereas heavy reliance on technologies, limited usability, or use of poorly designed technology can create negative feelings such as frustration, disengagement, and lower motivation.

Digital learning environments have a significant impact on both cognitive processes and metacognitive processes. Students can enhance their cognitive comprehension and problem-solving skills through educational technologies that offer visualization and simulation and adaptive feedback capabilities (Lyu et al., 2026; Alenezi et al., 2025; Chu et al., 2025). Digital dashboards and analytics-based feedback systems now provide more support for metacognitive skills that include self-monitoring, planning, and evaluation (Hizam et al., 2023; Dong et al., 2025; Huang et al., 2025). However, over-dependence on digital technology can hinder the processing of the information on a deeper level, and lead to the consumption of information in a passive way. This hints that the cognitive effects of digital transformation are determined by the design and implementation of technology in a learning process.

Self-regulation serves as a fundamental psychological aspect that digital transformation affects. Students in online and blended learning environments must take responsibility for their time management, goal setting, and their learning strategy control (Qiao et al., 2026; Zhang and Chen, 2026; Jiang et al., 2026). Digital tools can support these processes through structured learning pathways, reminders, progress monitoring, and adaptive feedback. Conversely, poorly regulated technology use may contribute to procrastination, problematic digital use, distraction, and diminished learning discipline, particularly among students with limited self-regulatory skills.

At the institutional stage, it is essential to observe that the efficiency of digital transformation is also determined by more general educational and organizational elements such as institutional leadership, policy frameworks, teacher preparedness, competence with technology, technological infrastructure, as well as organizational support (Moodley and Kern, 2026). The significance of these contextual variables lies in the fact that digital transformation can occur successfully only when certain technological, educational, and institutional environments are provided for it.

While these domains are related to one another in theory, previous studies have mostly investigated these fields separately. Students’ motivation and engagement in learning are believed to influence their willingness to participate in digital learning. Moreover, learners’ cognitive skills, self-regulation abilities, and metacognitive processes have a decisive impact on how efficiently students utilize digital resources. Likewise, the institutional and educational system factors may play a significant role in either promoting or inhibiting these student outcomes. Hence, the above-mentioned domains can be seen as components of the complex phenomena of wider psychological and educational implications of digital changes.

Nonetheless, the current literature shows contradictory results in this area. There are differences found according to study design, characteristics of population including socio-economical factors, level of education, cultural context, type of technology exposure, and implementation of study measures. Some of the provided studies show significant increases in terms of engagement, motivation, cognitive performance, or self-regulated learning but other ones report minimal impact or even decrease in motivation and digital well-being (Hizam et al., 2023; Jiang et al., 2023; Tu et al., 2026). Earlier assessments often emphasized technologies or the groups studied or outcomes achieved, so it is unclear whether there was enough evidence for a solid conclusion about the relationship among engagement, motivation, cognition/metacognition, self-regulation, and other factors linked to institutions or education systems. Furthermore, the variability of digital technologies and education settings makes it difficult to generalize findings attributed to “digital transformation.”

Thus, the primary gap that the current review attempts to fill is the absence of a systematic and theory-based synthesis of the stated five domains, which would examine them through the lens of digital transformation in education while considering the specifics of technologies, populations, educational contexts, research designs, and results. The mentioned five domains were determined beforehand as the key analytical domains of the current review and were chosen for the analysis because they concern various aspects of learners and educational contexts that can be affected by digital transformation.

The meta-analytic approach exists as the necessary method to combine data from multiple studies, which will create accurate assessment results about digital transformation impacts in educational settings. The meta-analysis process enables detect common patterns while studying multiple contexts through standardized statistical methods, which helps them determine study differences and the presence of publication bias. Nevertheless, because the selected technologies and related learning contexts are diverse, the aggregated results provided in this review are not regarded as an indication of the impact of one specific type of digital intervention. Instead, they signify the general trend of information across many different types of educational reforms involving digital applications.

Consequently, the systematic review and meta-analysis target the several questions given below: (1) What is the general effect of educational digital transformation on learning engagement and academic motivation? (2) What is the relationship of digital transformation with cognitive and metacognitive processes? (3) What is the correlation of the digital transformation with students’ self-regulated learning? (4) What are the institutional and educational system factors impacting implementation and effectiveness of digital transformation? and (5) Are there differences in these effects in relation to the population/educational context/technology/intervention/study design/outcome characteristics?

## Methodology

### Eligibility criteria

Studies were considered for inclusion if they provided quantitative information related to the effect of any form of educational digital transformation or technology-supported educational interventions on psychological or educational outcomes within an educational context. For the purpose of this review, educational digital transformation was considered a comprehensive transformation of educational activities and processes brought about through the use of digital technologies, platforms, tools, or technology-based practices that enhance or change the delivery of education, learning and assessment activities, communications, learner involvement, and administration functions. Technology-related tools under consideration included, but were not limited to, e-learning, online and blended learning, LMS, virtual or simulated classrooms, computer-based education, multimedia education, mobile learning, gamification, AI-supported education, learning analytics, and various other approaches enabling education with the use of technologies. In this study, the mentioned practices were analyzed as variants or components of educational digital transformation rather than equivalent interventions.

The population affected was those individuals employed in conventional educational environments such as education institutions, tertiary educational institutions, vocational institutions, and other formal learning environments. Studies were included if they should study how either digital technologies or digital transformations impact on educational learning in the context of a learning environment. Therefore, the studies that focused exclusively on workplace digital transformation, the use of technologies not related to education, smartphone addiction or nomophobia in the absence of education, or conceptual studies without any quantitative empirical data have been excluded.

Eligible studies were required to examine at least one of five predefined analytical domains: (1) learning engagement, (2) motivation and affective psychology, (3) cognitive and metacognitive psychology, (4) self-regulation and learning psychology, or (5) institutional and educational system factors. The population affected was those individuals employed in conventional educational environments such as education institutions, tertiary educational institutions, vocational institutions, and other formal learning environments. Studies were included if they should study how either digital technologies or digital transformations impact on educational learning in the context of a learning environment. Therefore, the studies that focused exclusively on workplace digital transformation, the use of technologies not related to education, smartphone addiction or nomophobia in the absence of education, or conceptual studies without any quantitative empirical data have been excluded.

Studies were determined to meet eligibility criteria by having the available presence of comparison condition or intervention contrast, including traditional or classic education, non-digital education, regular education practice, reduced use of the digital intervention, another form of education. Experimental and non-experimental studies, including cohort analysis, cross-sectional study, case–control study, and other observational studies were also eligible provided they had enough quantitative data to derive an effect size. Considering that the methodological variation between study designs of different nature is expected, study design was taken into account during data collection, risk of bias assessment, and during sensitivity analyses.

To be excluded from our study, a publication had to meet at least one of the exclusion criteria, which comprised of the following: the study had no education context, it did not directly assess eligible technology or process of transformation; the report did not include at least one of the qualifying psychological, educational, or institutional outcomes; the study was qualitative, editorials, commentaries or protocols; or the information was insufficient to compute effect sizes. How are populations, educational contexts, technologies, study designs, and outcome characteristics represented across the included evidence.

### Information sources

The initial database search identified 1,200 records, including PubMed (*n* = 223), Web of Science (*n* = 340), Embase (*n* = 222), Cochrane Library (*n* = 150), and Google Scholar (*n* = 265). All references of the selected articles and systematic reviews were further examined by hand. The search covered all the studies that have been published until the last day of the search preceding data analysis without any limitation to the publication date.

### Search strategy

A comprehensive search strategy incorporating controlled vocabulary, database-specific indexing terms, free-text terms, and Boolean operators was developed and adapted for each database. The core search strategy was:

*(“digital transformation” OR “educational technology” OR “e-learning” OR “online learning” OR “digital learning” OR “learning management system” OR “virtual classroom” OR “multimedia learning” OR gamification OR “artificial intelligence” OR “learning analytics”) AND (“learning engagement” OR motivation OR “academic motivation” OR “self-regulation” OR cognition OR metacognition OR “psychological outcomes” OR “learning outcomes”) AND (education OR student* OR learner* OR school* OR universit* OR “higher education”)**.

Search strategy was modified according to the needs of indexing for every particular database. Only peer-reviewed articles in English language were searched without any date limits. Complete database-specific search strategies are provided in the Supplementary Material to facilitate reproducibility. The final literature search was conducted on [20 March 2026].

### Data extraction procedure

Data extraction was performed using a standardized data-extraction form based on established systematic-review methodology and the Cochrane Handbook for Systematic Reviews of Interventions. The extracted variables included study characteristics, publication year, country, educational setting, participant characteristics, sample size, study design, digital technology or intervention characteristics, comparator or exposure contrast, outcome domain, measurement instruments, effect estimates, confidence intervals, and statistical information required for effect-size calculation.

The extracted data were checked against the original publications to minimize transcription and extraction errors. Where necessary, effect sizes were calculated from reported statistical information, including frequencies, contingency tables, means and standard deviations, correlation coefficients, regression estimates, and test statistics, using established meta-analytic conversion procedures.

No automated data-extraction software was used. Manual extraction was used because the included studies reported heterogeneous study designs, outcomes, statistical measures, and reporting formats. Title and abstract screening, full-text eligibility assessment, data extraction, effect-size calculation, and risk-of-bias assessment were conducted by the author. Uncertainties were resolved through re-evaluation against the predefined eligibility criteria.

### Data variables

The five primary outcome domains were defined *a priori* as: (1) learning engagement, (2) motivation and affective outcomes, (3) cognitive and metacognitive processes, (4) self-regulation and learning-related psychological processes, and (5) institutional and educational system factors.

Learning engagement included behavioral, emotional, and cognitive engagement. Motivation and affective outcomes included academic motivation and related affective responses. Cognitive and metacognitive outcomes included cognition, information processing, problem-solving, planning, monitoring, and evaluation. Self-regulation outcomes included goal setting, time management, learning-strategy control, monitoring, and related self-regulatory processes. Institutional and educational system factors included institutional support, technological infrastructure, organizational readiness, digital integration policies, teacher readiness, and learning-environment characteristics.

The five domains were specified before data synthesis and served as the conceptual framework and prespecified analytical subgroups of the review.

Secondary variables included study design, educational level, country, participant characteristics, sample size, technology or intervention type, comparator, duration of intervention or exposure, measurement instrument, and measurement reliability.

### Risk of Bias evaluation

The methodological quality of the included studies was assessed using a structured risk-of-bias approach addressing selection bias, measurement bias, confounding, attrition where applicable, and selective reporting. Studies were classified as having low, moderate, or serious risk of bias according to predefined criteria.

Risk-of-bias assessments were based on the reported study methodology, participant selection, outcome measurement, handling of confounding, completeness of outcome data, and reporting practices. The assessments were rechecked against the original publications before final classification. Risk-of-bias judgments were incorporated into sensitivity analyses and considered when interpreting the certainty and robustness of the evidence.

### Effect measures

Odds ratios (ORs) with corresponding 95% confidence intervals (CIs) were used as the common effect measure for the quantitative synthesis. This common metric was selected because a substantial proportion of the included studies reported dichotomous outcomes or statistical information that permitted valid conversion to ORs.

Where different effect measures were reported, including risk ratios, continuous outcomes, correlations, or other statistical estimates, the author converted them to ORs only when an established statistical conversion was possible and sufficient information was available. The original effect estimate and the corresponding converted estimate entered into the meta-analysis were recorded to ensure traceability from the published study to the pooled analysis. Studies for which a valid conversion could not be performed were not artificially transformed and were excluded from the relevant quantitative synthesis.

### Synthesis procedures

The studies included were synthesized in line with five predetermined outcome domains. The varied inclusion of technologies, populations, educational environments, study designs, comparators, and outcome measurements in the evidence base allows for interpreting the pooled estimates as indicative of the general evidence pattern across the types of digital educational transformation considered rather than of the impact of one consistent intervention.

Where required, reported statistics were transformed into ORs before pooling. Quantitative synthesis was performed using a random-effects model with inverse-variance weighting because substantial methodological and contextual heterogeneity was expected across the included studies.

Statistical heterogeneity has been measured using the Cochran Q-test and I^2^ statistic. While I^2^ is the primary statistic utilized in measuring between-study heterogeneity, Cochran’s Q statistic provides additional information on heterogeneity. Differences in study population, intervention type, educational environment, study design, and outcome measure must also be considered.

Publication bias and small-study effects were assessed using funnel plots and Egger’s regression test when at least 10 studies were available for a quantitative synthesis. The statistical analyses were performed using Review Manager (RevMan) version 5.4 and R version 4.3.1 using the meta and metafor packages.

### Reporting Bias assessment

The evaluation of the possibility of reporting bias as well as the small study effect was done by the author using both Egger’s regression and funnel plot symmetry wherever there were enough studies present. These evaluations were conducted for the overall synthesis and the author also did it for separate outcome domains where there was enough number of studies available. Funnel plot asymmetry was interpreted with caution since it could also point to heterogeneity among the studies in addition to any issues relating to publication bias.

### Certainty assessment

The certainty of evidence was evaluated using a structured GRADE-informed approach performed by the author, considering risk of bias, inconsistency, indirectness, imprecision, and publication bias for each major evidence domain. The certainty was evaluated separately for the five predetermined domains and ranked according to the levels of certainty high, medium, or low based on the strength, clarity of the available evidence. The evaluation also factored into considerations the variance in methodologies and context used in the pupil studies.

### Study selection

#### Selection process

The study selection process followed the PRISMA 2020 guidelines and included title and abstract screening followed by full-text assessment. All retrieved records were screened against the predefined eligibility criteria. Potentially relevant articles were subsequently assessed in full text. Eligibility decisions were based on the predefined inclusion and exclusion criteria, and any uncertainty was resolved by re-evaluating the study methodology, population, intervention or exposure, comparator, and outcome characteristics.

The initial database search identified 1,200 records, including PubMed (*n* = 223), Web of Science (*n* = 340), Embase (*n* = 222), Cochrane Library (*n* = 150), and Google Scholar (*n* = 265). After removal of 560 duplicate records, 640 unique records remained for title and abstract screening. Following this screening, 460 records were excluded because they did not meet the predefined eligibility criteria.

A total of 180 reports were sought for full-text retrieval. Additional retrieval attempts were made using alternative accessible sources and institutional or library routes. Of these, 120 reports remained unavailable, leaving 60 reports for full-text eligibility assessment. Fifteen full-text reports were excluded because of ineligible study design (*n* = 5), inappropriate population (*n* = 6), or inappropriate intervention/exposure (*n* = 7). The final quantitative synthesis included 42 studies that met all predefined eligibility criteria (Figure 1). The high number of unavailable reports was considered when interpreting the completeness and generalizability of the evidence base.

**Figure 1:**
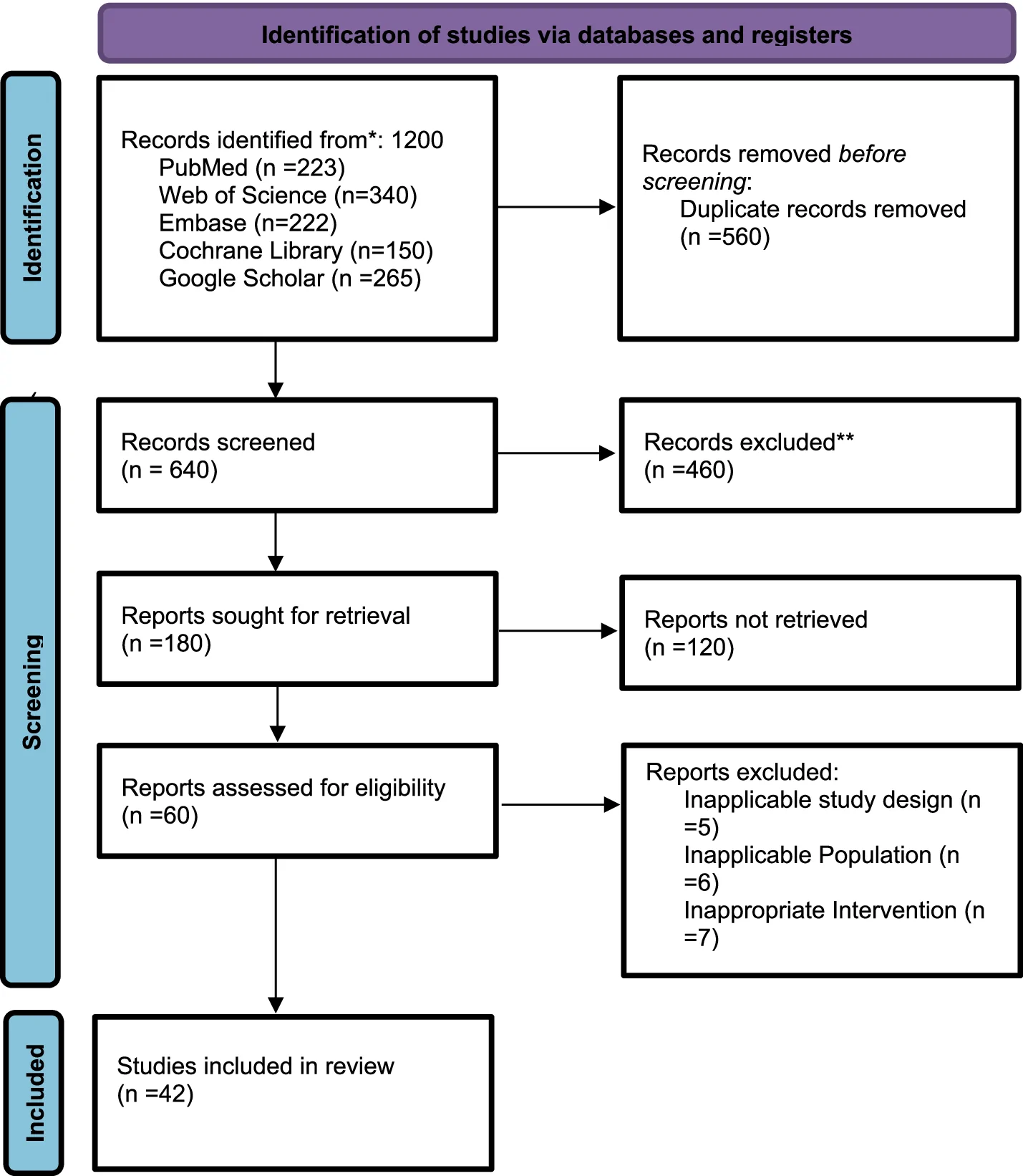
PRISMA flow chart of study selection.

#### Characteristics of the studies

This systematic meta-analysis was conducted on 42 global studies examining digital transformation in education and its psychological outcomes, including learning engagement, motivation, cognitive/metacognitive processes, self-regulation, and institutional factors. The studies originated from multiple nations (Figure 2).

**Figure 2:**
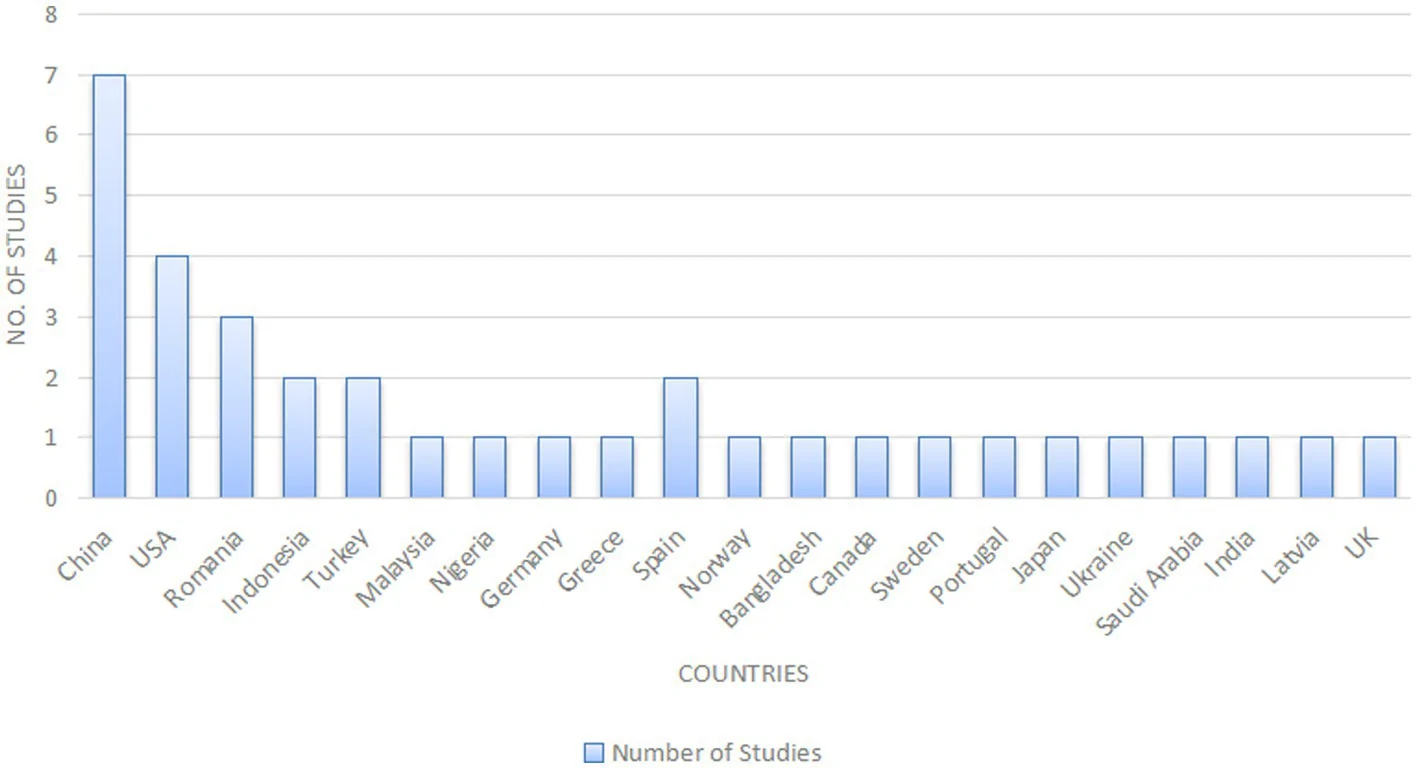
Studies by country (frequency distribution).

#### Study design distribution

The study used 10 different methods, which included Structural Equation Modeling (SEM) as the second most common design after quantitative studies, which reached 10 times of usage (*n* = 10), and used Structural Equation Modeling (SEM) as their testing method to examine the relationship between digital learning variables and psychological outcomes. The study used a cross-sectional design and mixed-method design to achieve equal representation of two research methods, which produced descriptive results together with data that combined qualitative and quantitative elements. Experimental studies (*n* = 3) and quasi-experimental designs (*n* = 1) provided evidence about how digital interventions cause particular effects, while the four studies on scale development and validation established reliable and valid psychological measurement instruments. The research field used two cognitive modeling studies, one longitudinal study, one ecological study, and one learning analytics study to achieve advanced analytical methods, which also provided time-sensitive perspectives, thereby enhancing the research study’s methodological rigor and evidence base through their combined contributions (Figure 3).

**Figure 3:**
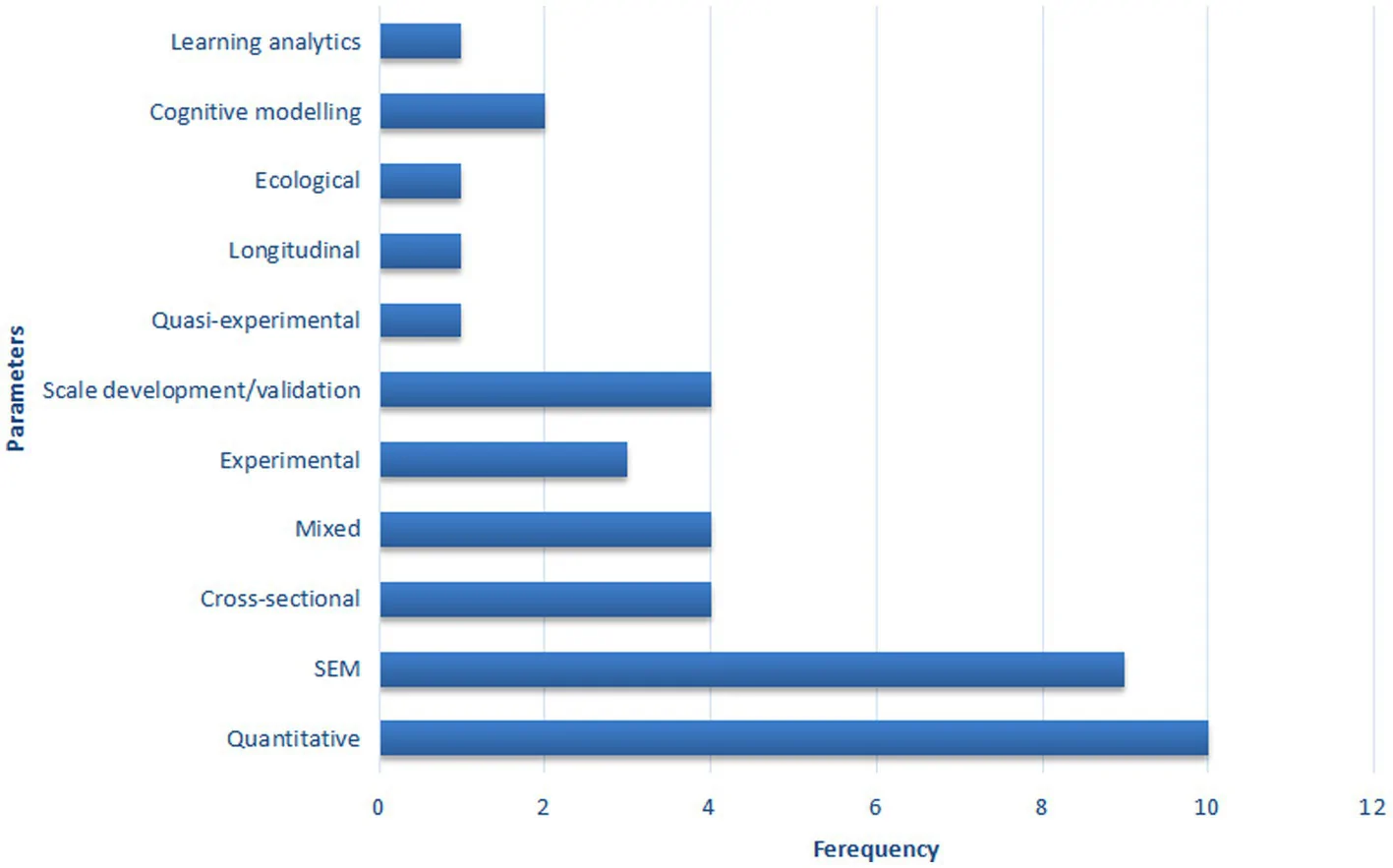
Study design distribution.

#### Sample size distribution

The research studies showed high sample size differentials because researchers studied digital transformation in various research environments and used different population groups. The most common sample size range was 300–400 participants (*n* = 12), followed by 200–300 (*n* = 10) and 400–500 (*n* = 10), indicating a strong concentration of medium-sized empirical studies. The research studies used smaller sample ranges of 50–150 and 100–200 with equal frequency (*n* = 4 each) because researchers conducted exploratory research or developed measurement scales. The larger studies included 500–600 participants (n = 4), 700–1,000 participants (*n* = 2), and only one study exceeded 1,000 participants. The distribution shows that most studies have moderate research power, and they maintain equal study representation among different educational environments (Figure 4).

**Figure 4:**
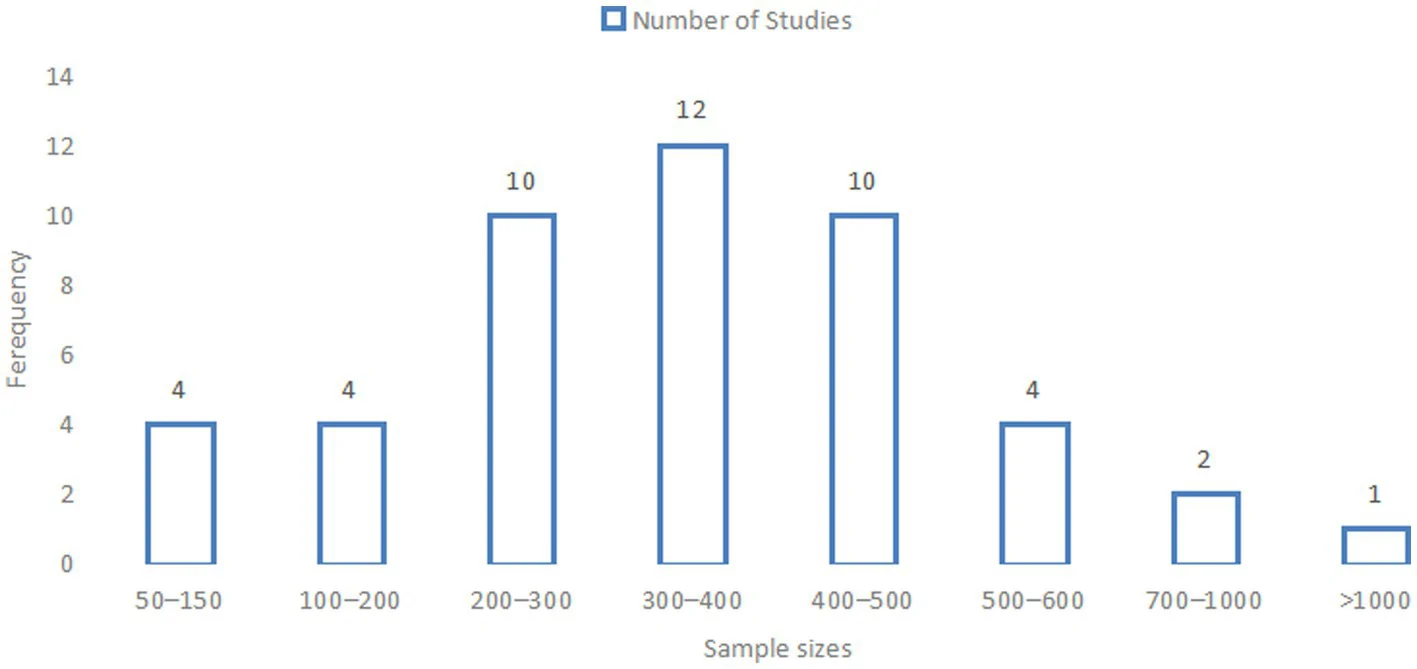
Sample size categories.

#### Population type distribution

The included studies covered a wide range of population groups, showing how digital transformation in education applies to different educational settings. The majority of studies focused on students (*n* = 28), because researchers considered students as their main subjects for studying learning engagement and psychological outcomes. The research included three studies, which focused on teachers and faculty members as teaching and learning subjects, while three studies combined student and faculty samples to provide teaching and learning insights. The study population included three groups of children, two groups of nursing students, two groups of employees or professionals, one group of academic workforce, one group of decision makers, and one group of educators. The research findings achieve greater external validity through their multiple educational and professional contexts, which study different groups of people (Figure 5).

**Figure 5:**
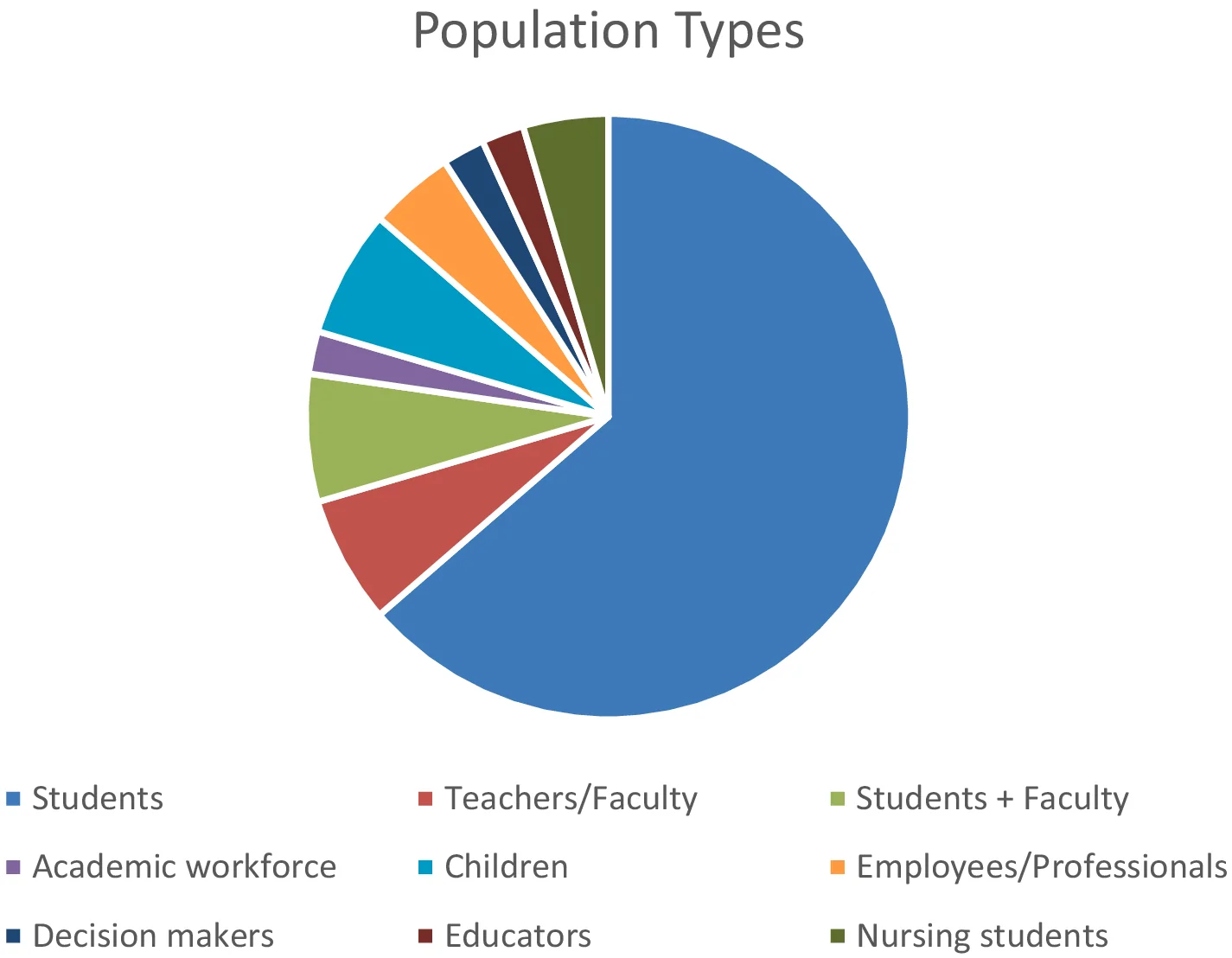
Population type distribution.

#### Data collection tools

The studies employed numerical data collection methods, which showed a preference for self-reported data through questionnaires and surveys, which researchers used in 32 cases. The four studies used mixed-method approaches, which combined different research tools to produce richer data and result in more precise research findings. The advanced digital data sources included Learning Management System (LMS) logs and platform analytics, which provided two sources of data that demonstrated how learners interacted with educational content. The study used three assessment methods, which included one interview, one cognitive test, and one behavioural scale to gather both qualitative data and performance measurements. The results show that researchers prefer to use structured survey methods, which they combine with new learning analytics technologies for their studies (Figure 6).

**Figure 6:**
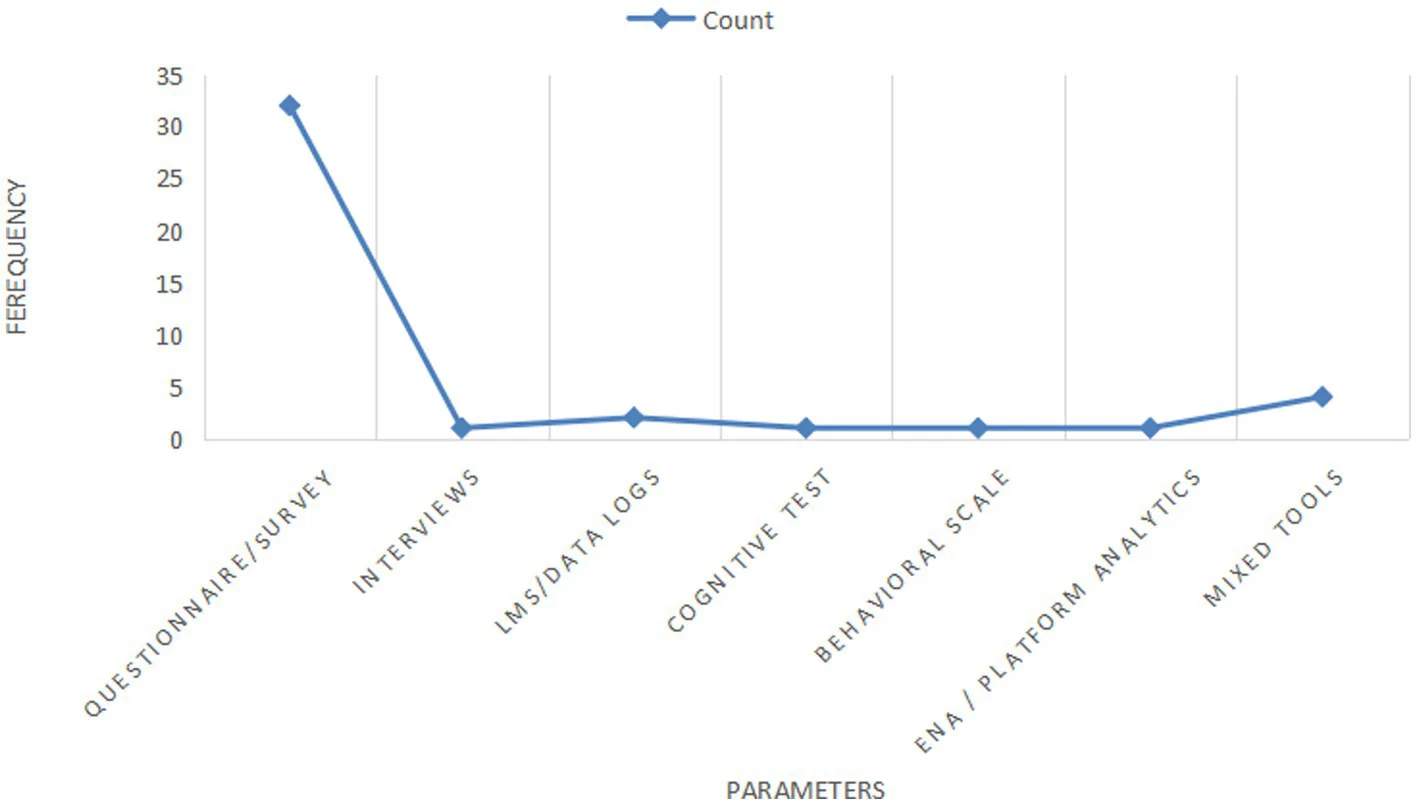
Data collection tools.

#### Key variables frequency

The meta-analysis included studies that investigated various psychological and educational factors that the digital transformation affects in educational settings. Learning engagement emerged as the most frequently investigated construct (*n* = 10), which shows its importance for educational results. The research studied digital transformation through six separate studies, which used it as their main intervention method. The studies assessed three different outcomes, which included cognitive results, self-efficacy, and digital skills or competencies, with four studies dedicated to each outcome. The studies treated self-regulation and teacher support or roles as essential elements that needed exploration through research. The research explored additional variables through gamification or games (*n* = 2) and technostress or nomophobia (*n* = 2) and leadership or resilience or AI-related constructs (*n* = 2), which show growing researcher interest towards emotional experiences and technology-based educational processes (Figure 7).

**Figure 7:**
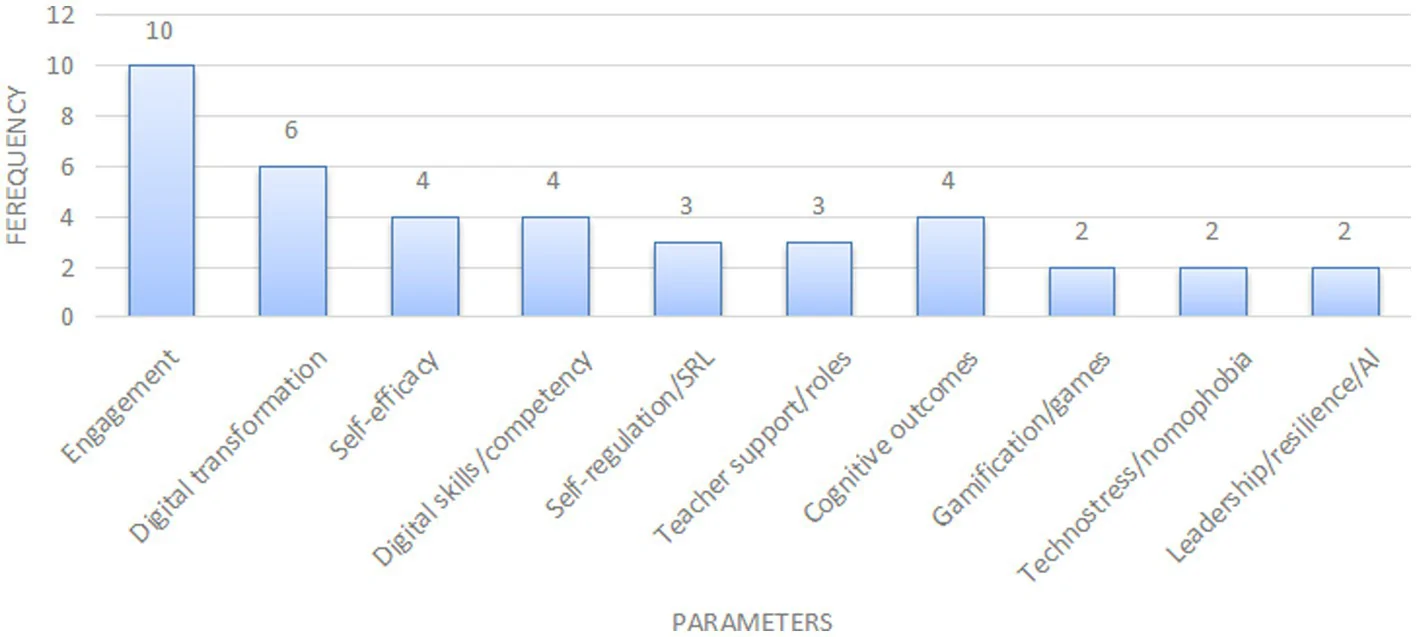
Key variables frequency.

### Statistical analysis methods

The studies that were conducted used various statistical methods to analyse digital transformation in education because researchers wanted to study the topic through different research methods. The researchers used regression analysis as their primary method, which they applied in 12 studies, while they used Structural Equation Modelling as their second most common method, which they applied in 11 studies to investigate how different variables interact with one another. The four studies used mixed methods research to combine their qualitative and quantitative results for a better research understanding. The three studies used experimental and mediation analysis methods to establish cause-and-effect relationships and evaluate different research pathways. Three studies used Confirmatory Factor Analysis (CFA) together with other psychometric methods to test their measurement tools. The research used two modelling techniques, two correlation analysis methods, one longitudinal study, and one thematic analysis method to build a diverse and methodologically complex evidence base (Figure 8).

**Figure 8:**
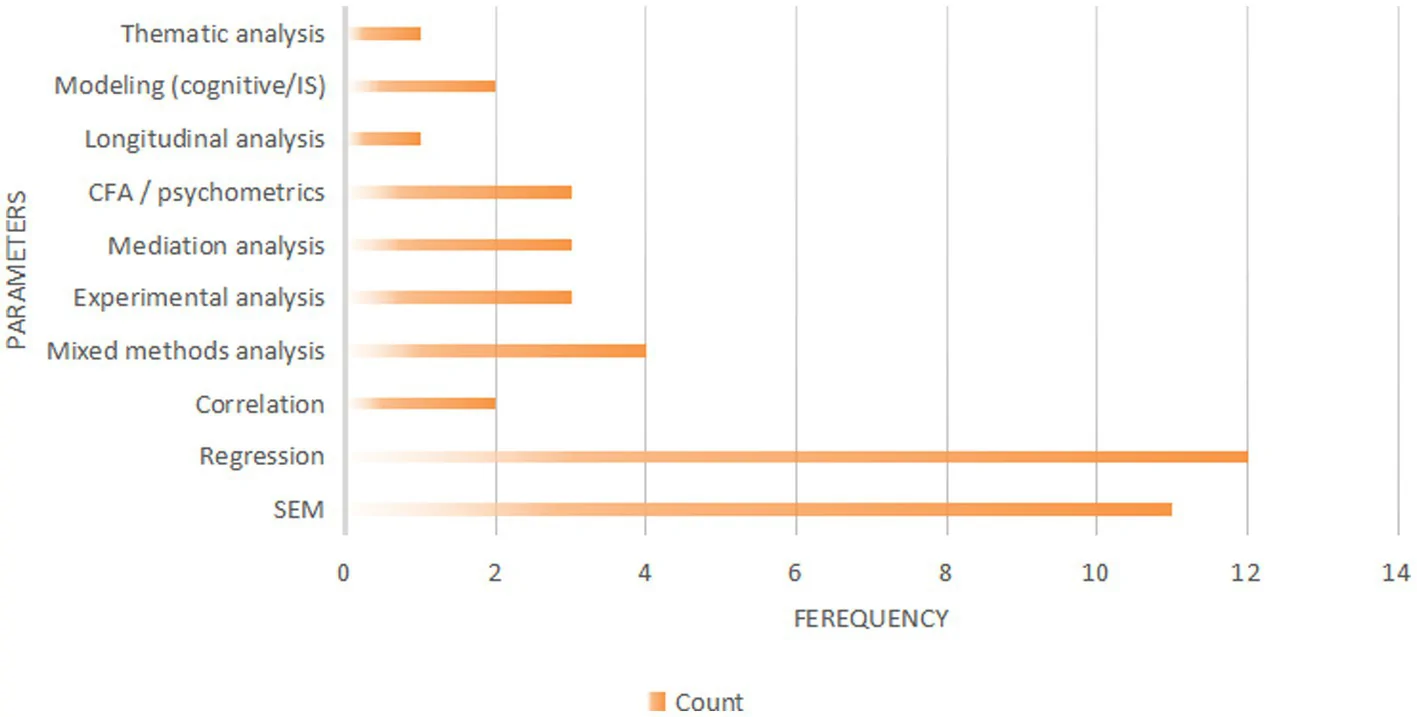
Statistical analysis methods.

### Sampling techniques

The studies included in this meta-analysis employed a variety of sampling techniques, which showed different study design methods and data accessibility levels between studies. Convenience sampling was the most frequently used approach (*n* = 16), which showed researchers chose samples from educational settings that were easily accessible. Mixed sampling strategies were also common (*n* = 9), which used several methods to achieve better sample representation and increased data accuracy. Researchers used stratified sampling (*n* = 4) and purposive sampling (*n* = 3) to achieve targeted or subgroup representation, while random or national sampling methods (*n* = 3) enabled better generalisation results. Researchers used institutional or controlled sampling methods in two studies, while one study used each of voluntary online and classroom-based sampling methods. The distribution data shows that non-probability sampling methods dominate, while probability-based and structured methods exist to improve research quality (Figure 9).

**Figure 9:**
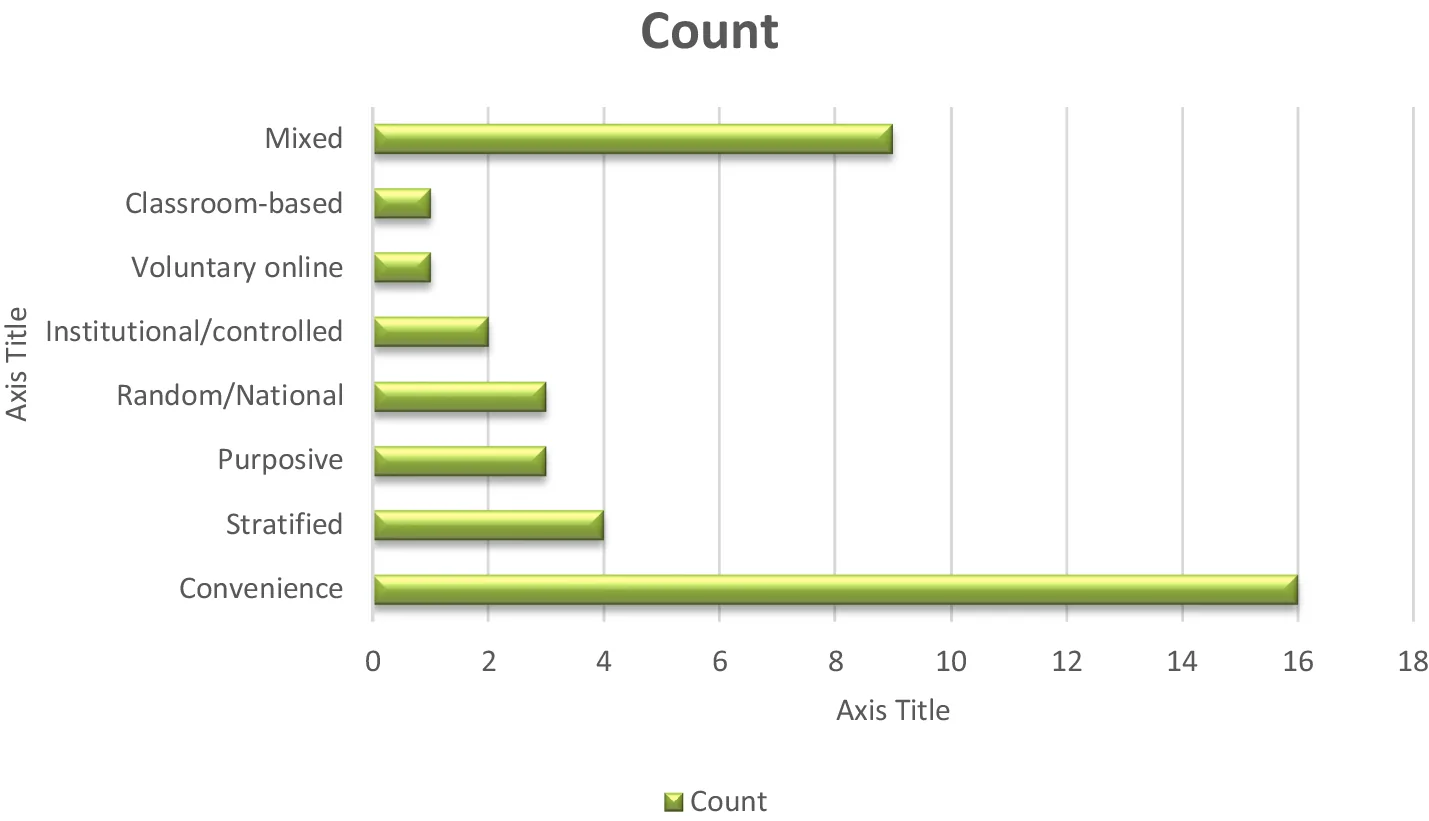
Sampling techniques.

### Research context

The study results show that digital transformation research can be applied to different educational environments and professional domains. The majority of studies were carried out in higher education or university environments (*n* = 27), highlighting the strong focus on tertiary-level learning contexts. Schools conducted 8 studies of primary and secondary education, while online learning platforms were studied in five studies, which shows how digital and blended learning environments have become more important. Healthcare education (*n* = 2), national survey-based studies (*n* = 2), and laboratory or controlled experimental settings (*n* = 2) provided more specialised contexts. The research included one study that focused on industry or firm-based settings and one study that examined education project-based research. The research results show that educational institutions, especially universities, serve as the main sites where researchers explore digital transformation in educational settings (Figure 10).

**Figure 10:**
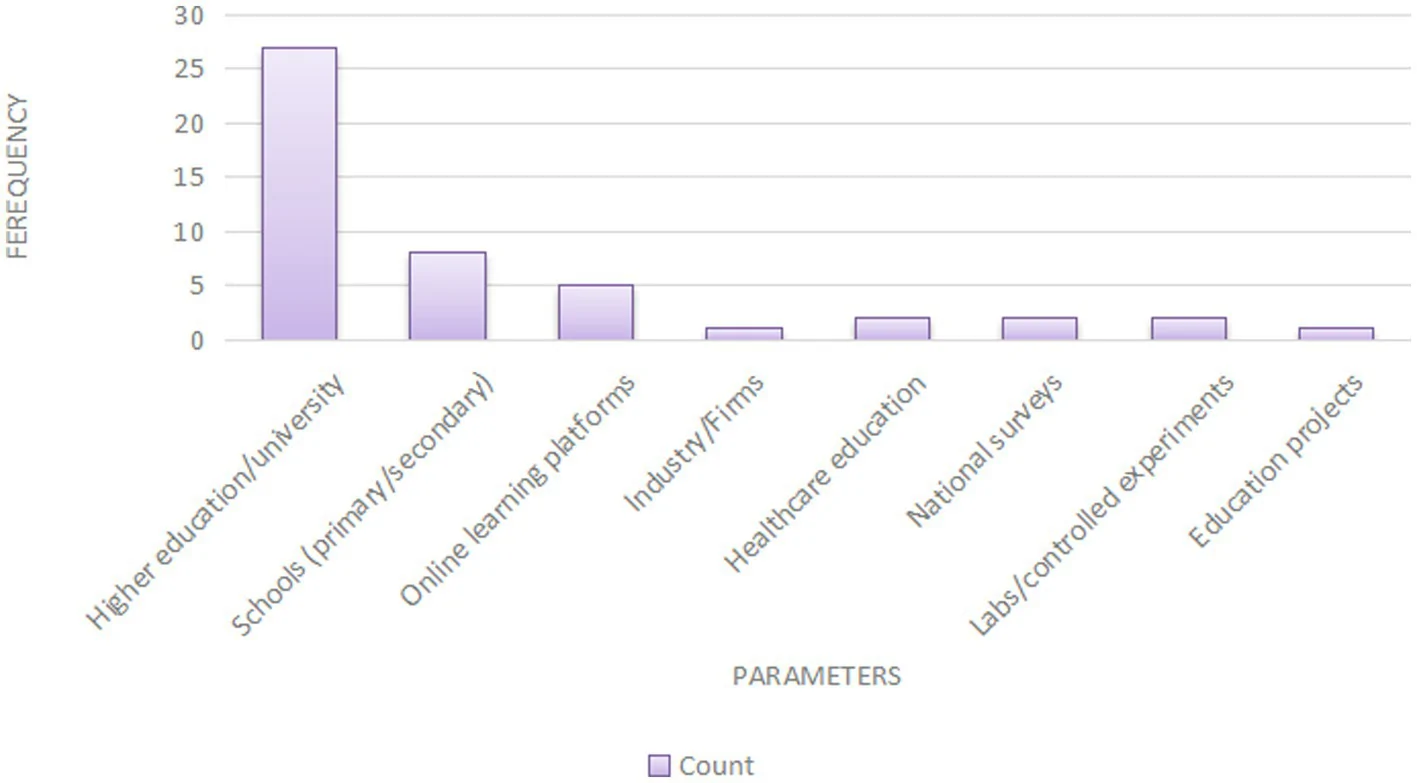
Research context / setting.

## Results

### Risk of Bias assessment

Most studies exhibited moderate bias risk because their research used cross-sectional designs and their participants provided self-reported data. The validated experimental studies and analytics-based research showed low risk of bias in three studies. The research identified two studies that had serious risk their primary focus was on conceptual or theoretical research. The evidence base demonstrates moderate methodological quality because it meets acceptable reporting standards (Table 1).

**Table 1:** Risk of bias assessment.

Study	Selection Bias	Measurement Bias	Confounding	Reporting Bias	Overall Risk of Bias
Niță and Guțu (2023)	Moderate	Moderate	Moderate	Low	Moderate
Olawumi and Onasanya (2025)	Moderate	Moderate	High	Moderate	Serious
Enachescu (2025)	Moderate	Moderate	Moderate	Low	Moderate
Hizam et al. (2023)	Low–Moderate	Moderate	Moderate	Low	Moderate
Aljanazrah et al. (2022)	Moderate	High	Moderate	Moderate	Serious
Packmohr and Brink (2023)	Moderate	Moderate	Moderate	Moderate	Moderate
Fatimah et al. (2026)	High	High	High	Moderate	Serious
Neagu and Vieriu (2025)	Moderate	Moderate	Moderate	Low	Moderate
Dong (2025)	Moderate	Moderate	Moderate	Low	Moderate
Li et al. (2024)	Moderate	Moderate	Moderate	Low	Moderate
Kaimara (2026)	High	High	Moderate	Moderate	Serious
Aspandi and Muttaqin (2025)	Moderate	Moderate	Moderate	Low	Moderate
Pepple (2022)	Moderate	Moderate	Moderate	Low	Moderate
Xu et al. (2025)	Low–Moderate	Moderate	Moderate	Low	Moderate
Jallali et al. (2026)	High	High	High	Moderate	Serious
Abubakarı et al. (2022)	Moderate	Moderate	Moderate	Low	Moderate
Jang et al. (2026)	Low	Moderate	Moderate	Low	Moderate
Artino and McCoach (2008)	Low	Low	Low	Low	Low
Bakır-Yalçın and Usluel (2024)	Moderate	Moderate	Moderate	Low	Moderate
Avcı and Ergün (2022)	Low–Moderate	Moderate	Moderate	Low	Moderate
Bergdahl et al. (2020)	Moderate	Moderate	Moderate	Low	Moderate
Barnard et al. (2009)	Low	Low	Low	Low	Low
Black and Deci (2000)	Low	Low	Low–Moderate	Low	Low–Moderate
Chan et al. (2021)	Moderate	Moderate	Moderate	Low	Moderate
Chen (2023)	Moderate	Moderate	Moderate	Low	Moderate
Cheng and Tsai (2011)	Moderate	Moderate	Moderate	Moderate	Moderate
Cho et al. (2017)	Low–Moderate	Moderate	Moderate	Low	Moderate
Coates (2010)	Low	Low	Low	Low	Low
Coelho et al. (2019)	Moderate	Moderate	Moderate	Low	Moderate
Dixson (2015)	Low	Low	Low	Low	Low
Elshami et al. (2022)	Moderate	Moderate	Moderate	Low	Moderate
Fan et al. (2021)	Low–Moderate	Moderate	Moderate	Low	Moderate
Feng et al. (2023)	Moderate	Moderate	Moderate	Low	Moderate
Gherghel et al. (2023)	Low–Moderate	Moderate	Moderate	Low	Moderate
Guo et al. (2023)	Low	Low–Moderate	Moderate	Low	Moderate
Nikitenko et al. (2022)	High	High	High	Moderate	Serious
Zhou (2024)	Moderate	Moderate	Moderate	Low	Moderate
Russell et al. (2020)	High	High	High	Moderate	Serious
Maslennikova et al. (2019)	High	High	High	Moderate	Serious
Palacios-Rodríguez et al. (2023)	High	High	High	Moderate	Serious
Raj et al. (2026)	Moderate	Moderate	Moderate	Low	Moderate
Veckalne and Tambovceva (2022)	High	Moderate	Moderate	Moderate	Serious

### GRADE evidence assessment

The GRADE framework was used to assess the total certainty of evidence which exists throughout the studies that were selected for evaluation. Group 1 (Digital Transformation, Leadership & Institutional Change) demonstrated moderate certainty because of its moderate bias risk and its moderate risk of inconsistency together with its low-to-moderate indirectness. Group 2 (Online Learning Engagement, LMS Use & Self-Regulation) showed moderate certainty, but researchers discovered more study inconsistencies than they had anticipated. Group 3 (Digital Pedagogy, Gamification & Educational Technology Interventions) received a low to moderate rating because of its moderate indirectness and its publication bias issues. The Digital Well-being, Addiction, Nomophobia & Psychological Impact research group showed low to moderate certainty because its research design contained high bias risk and moderate study inconsistencies. Group 5 (Cognitive Development, Metacognition & Digital Skills) showed low to moderate results because high imprecision existed within the study despite its low inconsistency. Group 6 (Engagement Theory, Measurement Tools & Conceptual Models) reached moderate certainty because its findings showed low bias risk and high study consistency but researchers found both indirectness and imprecision to be problem (Table 2).

**Table 2:** GRADE evidence assessment.

Evidence group	Risk of Bias	Inconsistency	Indirectness	Imprecision	Publication Bias	Overall certainty
Group 1: Digital transformation, leadership & institutional change	Moderate	Moderate	Low–Moderate	Moderate	Possible	Moderate
Group 2: Online learning engagement, LMS use & self-regulation	Moderate	Moderate–High	Low	Moderate	Possible	Moderate
Group 3: Digital pedagogy, gamification & educational technology interventions	Moderate	Low–Moderate	Moderate	Moderate	Possible	Low–Moderate
Group 4: Digital well-being, addiction, nomophobia & psychological impact	Moderate–High	Moderate	Low	Low–Moderate	Possible	Low–Moderate
Group 5: Cognitive development, metacognition & digital skills	Moderate	Low	Moderate	High	Possible	Low–Moderate
Group 6: Engagement theory, measurement tools & conceptual models	Low–Moderate	Low	High	High	Low–Moderate	Moderate

### Meta-analysis

The meta-analysis used a random-effects model, which applied inverse variance weighting to combine findings from five psychological and institutional research fields. Digital Transformation → Learning Engagement showed a significant positive effect (OR = 1.79, 95% CI: 1.68–1.91) with low heterogeneity and no publication bias. Motivation & Affective Psychology showed a significant effect (OR = 1.57), but results showed substantial heterogeneity (I^2^ = 80%). Cognitive & Metacognitive Psychology showed a strong significant effect (OR = 1.79), but Egger’s test showed results which might indicate publication bias. Self-Regulation & Learning Psychology (9 studies) found a significant effect which was weaker (OR = 1.66) but showed high variability. Institutional & Educational System Factors showed a significant positive effect (OR = 1.71) which showed low variability and no publication bias. Digital transformation brought psychological and institutional improvements to all domains, which exhibited different patterns of bias and subgroup variability (Table 3).

**Table 3:** Meta-analysis summary.

Group	No. of studies	Pooled OR	95% CI (Lower)	95% CI (Upper)	Overall Effect (*p*-value)	Heterogeneity	Publication bias (Funnel Plot)	Egger’s Intercept	Egger’s 95% CI	*t*-value	Egger’s *p*-value
Digital Transformation → learning engagement	21	1.79	1.68	1.91	< 0.05 (significant)	Not significant (low heterogeneity; effects consistent)	No evidence of publication bias	−3.22	−8.07 to 1.63	−1.301	0.208
Motivation and affective psychology	6	1.57	1.21	2.03	< 0.05 (significant)	Significant heterogeneity (*p* < 0.01; I^2^ = 80%)	No evidence of publication bias	29.3	0.77 to 57.83	2.013	0.114
Cognitive and metacognitive psychology	6	1.79	1.60	2.02	<0.05 (significant)	Not notable heterogeneity (uniform effects)	Potential publication bias detected	−14.11	−21.98 to −6.24	−3.512	0.025
Self-regulation and learning psychology	7	1.66	1.49	1.85	<0.05 (significant)	Significant heterogeneity (p < 0.01; I^2^ = 0%)	No evidence of publication bias	−0.23	−34.85 to 34.4	−0.013	0.9947
Institutional and educational system factors	4	1.71	1.48	1.97	<0.05 (significant)	Not notable variability (effects uniform in direction and magnitude)	No evidence of publication bias	8.64	2.35 to 14.92	2.694	0.115

### Digital transformation

Researchers examined a total of 21 separate research studies. The random effects model showed that the study results produced an odds ratio value of 1.79 which exists within the 95% confidence range of 1.68 to 1.91 and demonstrates statistical significance because of the *p*-value threshold which fell below 0.05. The studies showed no significant diversity in their results because all studies produced identical treatment effects. The funnel plot showed that publication bias did not exist, and Egger’s test showed no significant intercept irregularities (intercept: -3.22, 95% CI: −8.07 to 1.63, t = −1.301, *p* = 0.208). Digital transformation creates a consistent positive impact on learning engagement for multiple educational contexts, which research shows maintains its strength across different study methods and participant groups and teaching methods. The research demonstrates that digital tools including LMS platforms and gamification and AI-based learning systems and digital leadership practices serve as effective tools for enhancing student engagement. The evidence base maintains its strength through two factors, which include the absence of publication bias and heterogeneity, while it demonstrates that digital transformation serves as a fundamental factor that improves student engagement in contemporary educational systems (Figure 11).

**Figure 11:**
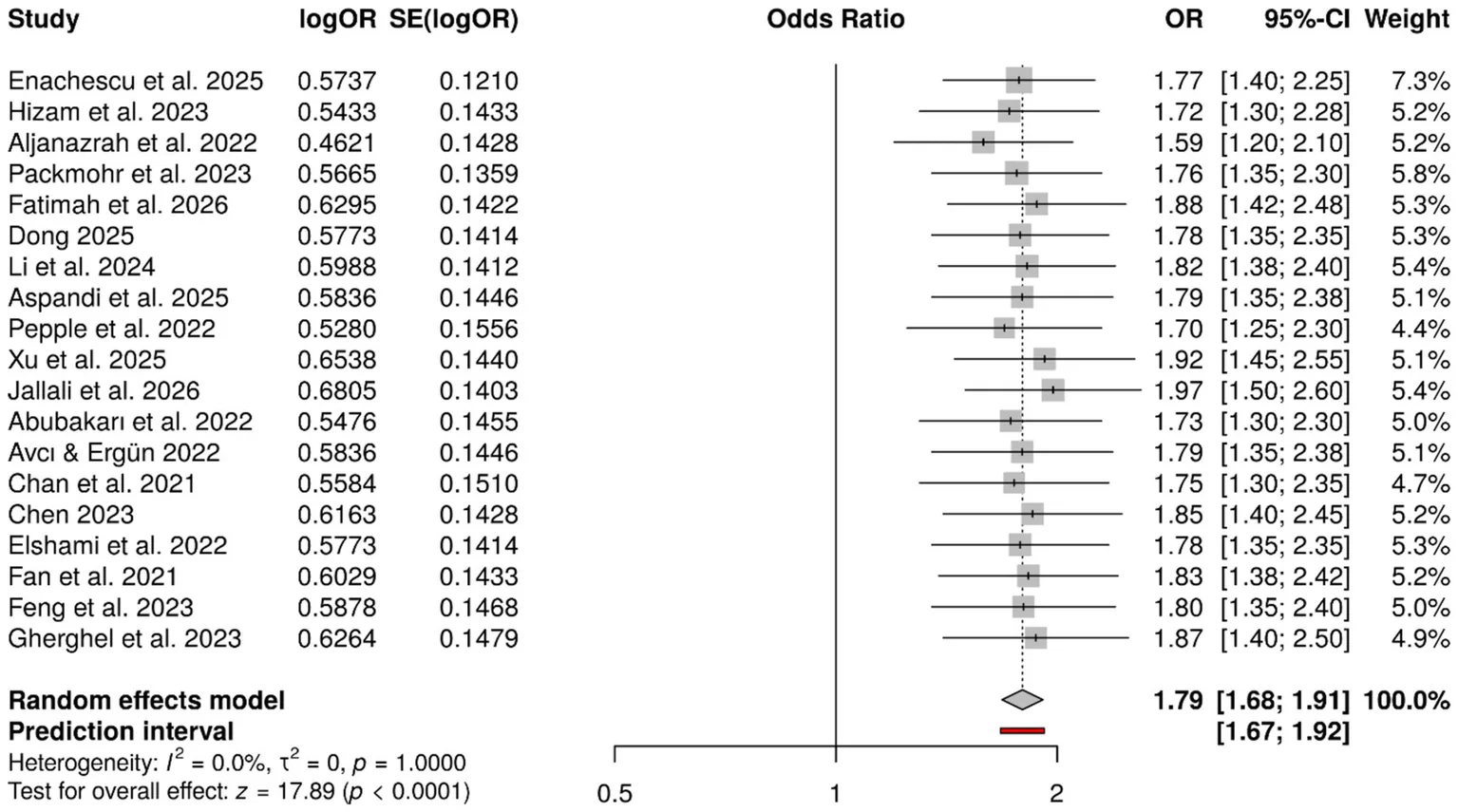
Forest plot of the studies about Digital Transformation.

### Motivation and affective psychology

The research involved examination of six distinct studies. The random effects model with inverse variance method showed an odds ratio (OR) of 1.57 which had a 95% confidence interval that extended from 1.21 to 2.03 and demonstrated a statistically significant effect because of the result with *p* value under 0.05. The analysis revealed significant heterogeneity which showed that effect sizes across studies displayed two distinct patterns of measurement under which they produced different results (*p* < 0.01; I^2^ = 80%). All evidence showed no publication bias, while Egger’s test results showed no statistical significance (intercept: 29.3, 95% CI: 0.77 to 57.83, t = 2.013, *p* = 0.114). The research results demonstrate that digital transformation creates a beneficial effect on both motivation and affective psychology which varies according to the type of used intervention. The results show that motivational and emotional responses to different pedagogical designs and learner contexts respond more acutely than engagement outcomes (Figure 12).

**Figure 12:**
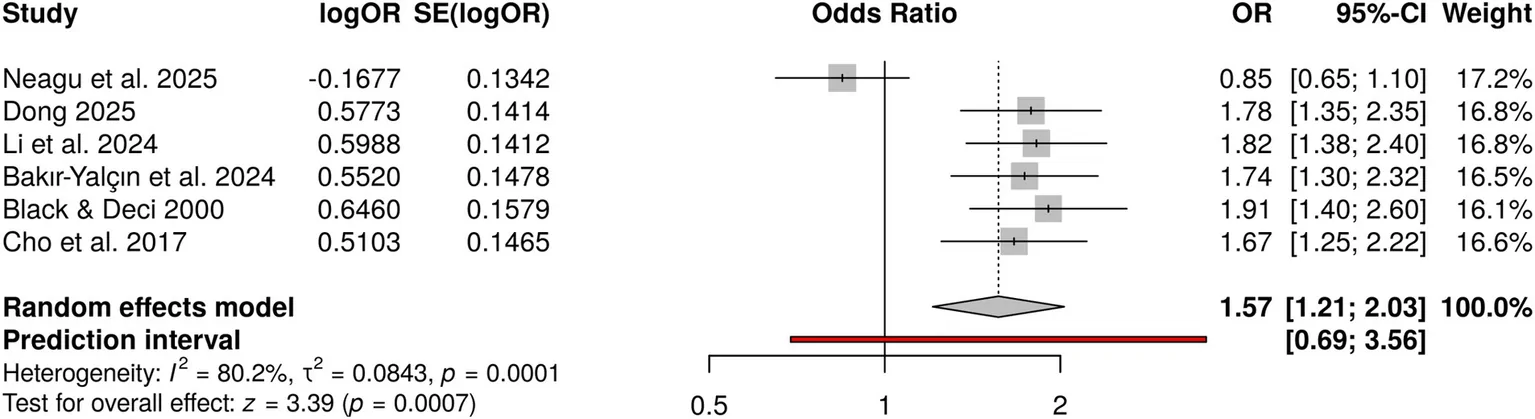
Forest plot of the studies about Motivation & Affective Psychology.

### Cognitive and metacognitive psychology

A total of six studies were examined in this research. The random effects model with inverse variance method produced an odds ratio (OR) which reached 1.79 and established a 95% confidence interval between 1.60 and 2.02 which demonstrated a statistically significant effect (*p* < 0.05). The research found no major heterogeneity because all studies showed identical effect sizes that were both consistent and stable. The results from Egger’s test showed publication bias that produced an intercept of −14.11 and a 95% confidence interval that ranged from −21.98 to −6.24 with a t value of −3.512 and a *p* value of 0.025 which indicated possible funnel plot asymmetry. The research results demonstrate that digital transformation has a beneficial impact on cognitive and metacognitive outcomes that show stable effects in all studies. The existing publication bias requires careful analysis of the results because it makes smaller and non-significant research studies less visible in the academic field that leads to an inflated effect size (Figure 13).

**Figure 13:**
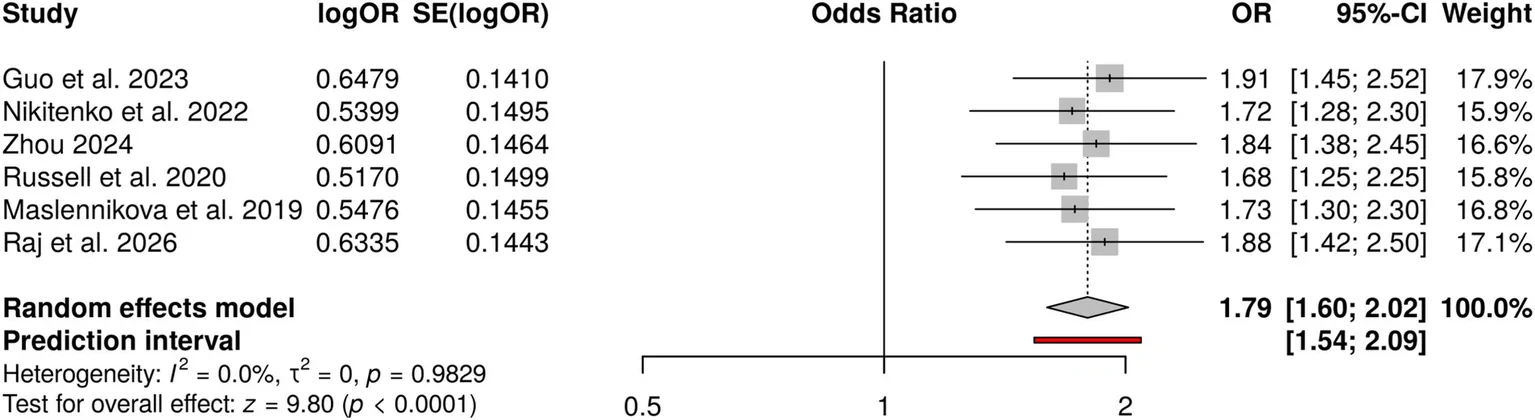
Forest plot of the studies about Cognitive & Metacognitive Psychology.

### Self-regulation and learning psychology

Seven distinct studies were used for analysis. The random effects model applied with the inverse variance method produced an odds ratio (OR) estimate of 1.66, which had a 95% confidence interval ranging from 1.49 to 1.85. This result showed statistical significance through a *p*-value less than 0.05. The study results revealed no significant heterogeneity because (*p* < 0.01; I^2^ = 0%). The analysis showed no publication bias, and Egger’s test results showed no statistical significance because the intercept measured −0.23 with a 95% confidence interval from −34.85 to 34.4, a t score of −0.013 and a *p* value of 0.9947. The research results demonstrate that digital transformation improves self-regulation together with learning-related psychological processes, which include self-efficacy and self-regulated learning and engagement development, while digital overuse behaviours such as nomophobia and smartphone addiction produce mixed results. The strong heterogeneity shows that results depend on specific situations, which are affected by learner traits, intervention methods and the design of digital learning environments (Figure 14).

**Figure 14:**
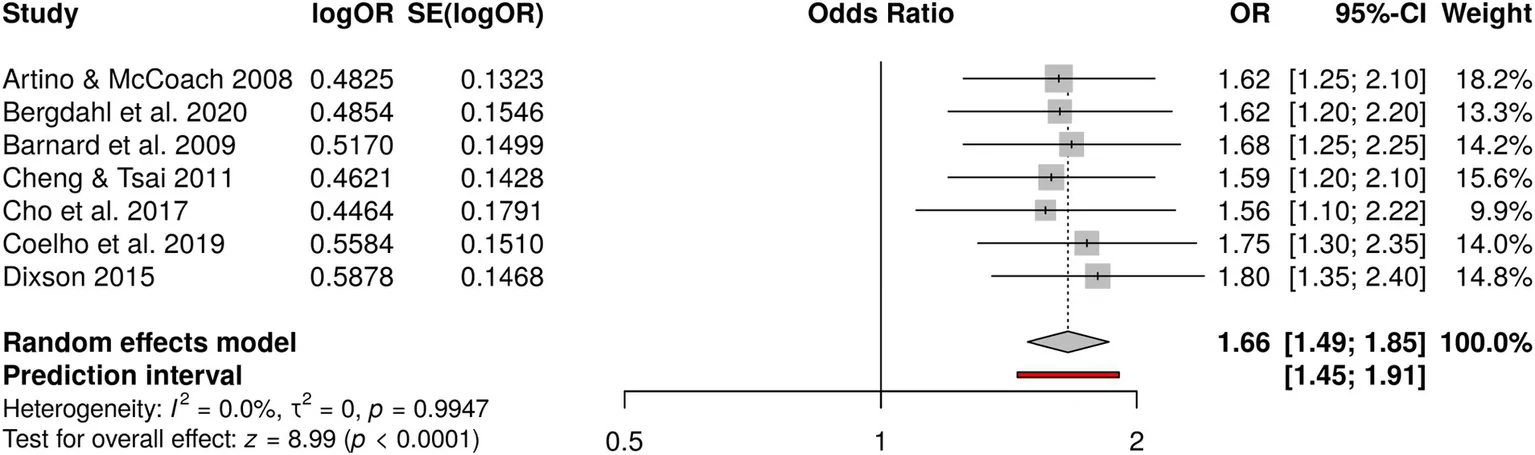
Forest plot of the studies about Self-Regulation & Learning Psychology.

### Institutional and educational system factors

The analysis includes a total of four studies. The random effects model with inverse variance method produced an odds ratio (OR) result which showed a 1.71 value, and this result reached 95% confidence interval which spanned from 1.48 to 1.97, and this result demonstrated a statistically significant effect because the *p*-value reached below the 0.05 threshold. The study results showed that effect sizes maintained their uniformity because both their size and direction remained constant throughout the research. The study results showed that no publication bias existed, and the analysis through Egger’s test found no evidence of funnel plot asymmetry with an intercept value of 8.64, which had a 95% confidence interval between 2.35 and 14.92 and a *t* value of 2.694, which produced a *p* value of 0.115. The research results demonstrate that institutional and educational system factors, which include digital transformation policies, competence development frameworks, communication skills enhancement and national engagement strategies, serve as fundamental elements that produce educational improvements. The research results demonstrate that system-level interventions serve as critical methods for boosting user participation, while these methods enable educational institutions to achieve lasting digital transformation (Figures 15, 16).

**Figure 15:**
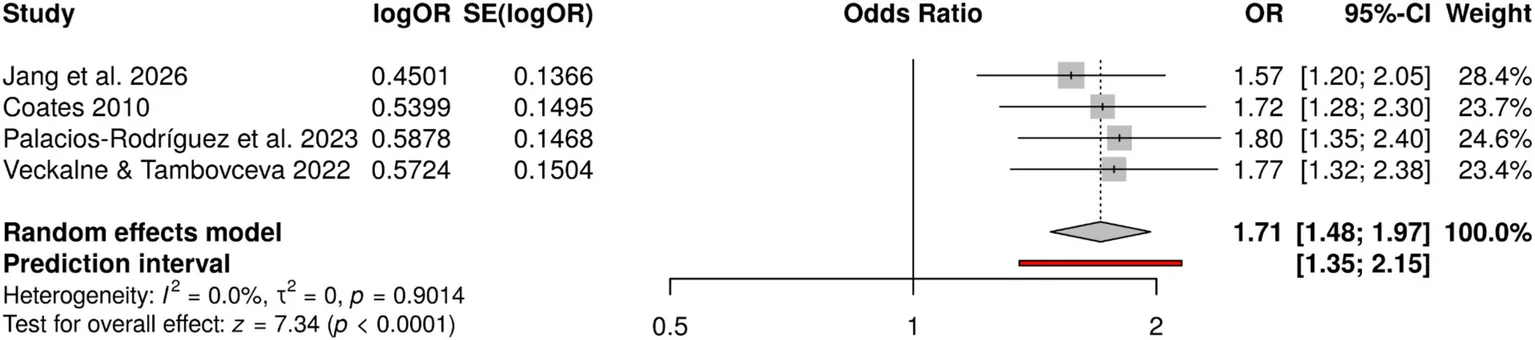
Forest plot of the studies about Institutional & Educational System Factors.

**Figure 16:**
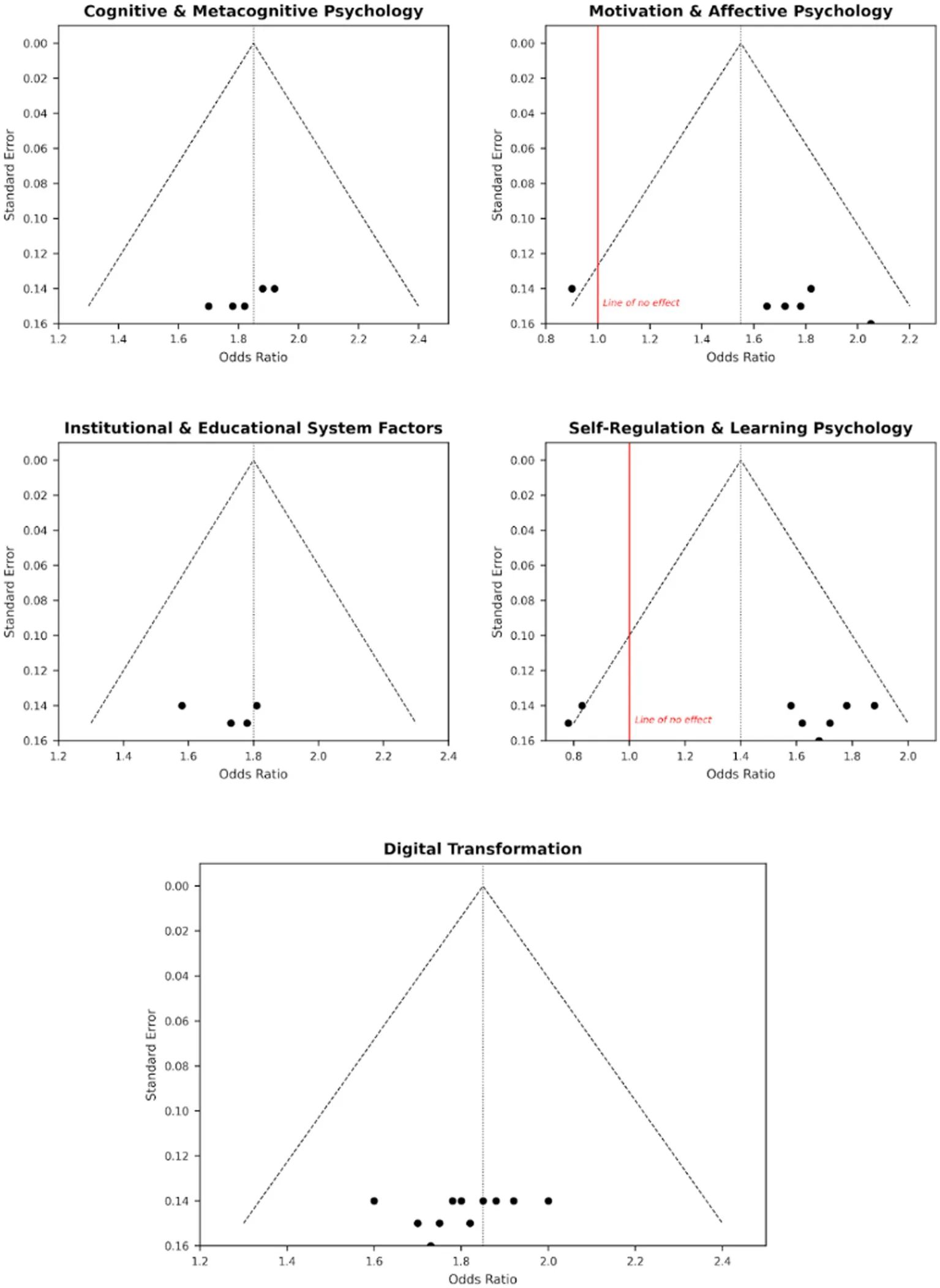
Funnel plot of the included studies.

## Discussion

This meta-analysis aimed to synthesize global evidence on the psychological perspectives of digital transformation in education, focusing on learning engagement, motivation, cognitive/metacognitive processes, self-regulation, and institutional factors. The meta-analysis synthesises 42 (Niță and Guțu, 2023; Olawumi and Onasanya, 2025; Enachescu, 2025; Hizam et al., 2023; Aljanazrah et al., 2022; Packmohr and Brink, 2023; Fatimah et al., 2026; Neagu and Vieriu, 2025; Dong, 2025; Li et al., 2024; Kaimara, 2026; Aspandi and Muttaqin, 2025; Pepple, 2022; Xu et al., 2025; Jallali et al., 2026; Picazo Rodríguez et al., 2024; Aarestad et al., 2023; Al-Mamun et al., 2023; Abubakarı et al., 2022; Jang et al., 2026; Artino and McCoach, 2008; Bakır-Yalçın and Usluel, 2024; Avcı and Ergün, 2022; Bergdahl et al., 2020; Barnard et al., 2009; Black and Deci, 2000; Chan et al., 2021; Chen, 2023; Cheng and Tsai, 2011; Cho et al., 2017; Coates, 2010; Coelho et al., 2019; Dixson, 2015; Elshami et al., 2022; Fan et al., 2021; Feng et al., 2023; Gherghel et al., 2023; Guo et al., 2023; Nikitenko et al., 2022; Zhou, 2024; Russell et al., 2020; Maslennikova et al., 2019; Palacios-Rodríguez et al., 2023; Raj et al., 2026; Veckalne and Tambovceva, 2022) research studies which investigate how digital transformation affects educational psychology through five main research areas, which include learning engagement, motivation together with emotion research, cognitive processes together with metacognitive processes, self-regulation, and institutional/educational system factors. The research results demonstrate that digital transformation produces beneficial results for both educational outcomes and psychological outcomes, but different domains show distinct levels of effect strength and effect reliability, which mark essential theoretical and methodological distinctions. The learning engagement domain showed the strongest and most stable effect, which resulted in an odds ratio of 1.79, together with low study variability and no signs of publication bias. Digital transformation tools, which include Learning Management Systems LMS AI-supported instruction, gamification and digital leadership strategies, lead to increased learner participation, together with behavioural involvement. The effect maintains its stability across various contexts because engagement functions as a responsive construct that technology integration produces through its visible and active behavioural manifestations. The results support current engagement theories, which see interactive digital environments as essential tools for maintaining learning attention and active participation during educational activities.

The researchers found that motivational psychology and affective psychology produced a moderate impact, which displayed unpredictable results (OR = 1.57) and showed high heterogeneity (I^2^ = 80%). The research shows that people react emotionally and motivationally to digital transformation based on specific conditions. The various elements of instructional design, together with learner autonomy, cultural expectations and digital pedagogy standards, create different educational results. The research shows that autonomy-supportive environments and gamified systems create positive effects on motivation, while poorly designed digital systems and excessive digital demands lead to negative effects on psychological health. The domain-specific inconsistency demonstrates that technological systems create motivation only when educators design educational activities, and students understand educational resources. The research identified self-regulation as the main factor that affected learning psychology, and self-regulation showed weaker effects than self-regulation, which produced an odds ratio of 1.66. Digital environments provide students with self-regulated learning (SRL) capabilities through their ability to monitor progress, receive adaptive feedback, and learn flexibly, yet these environments create difficulties, including excessive mental strain, interruptions and continuous digital fatigue. The presence of negative behaviour outcomes, which include nomophobia and smartphone addiction, makes it harder to understand digital transformation because it enhances and disrupts self-regulatory processes based on how people use technology and their personal characteristics. The process requires educational institutions to develop digital learning designs that create structured learning paths that enable students to control their learning activities.

The results from cognitive and metacognitive psychology research established a significant effect, which measured 1.79 as its odds ratio with only minimal study variation, but with the detection of publication bias. The literature currently faces a problem because studies tend to exaggerate their results when researchers fail to disclose their findings that prove to be neutral or unfavourable. Research demonstrates cognitive benefits through improved decision-making ability and better cognitive engagement and development of metacognitive skills, although publication bias affects the study results. Research should focus on transparency as its main objective, while including studies that show no results to enhance the trustworthiness of evidence. Educational institutions and their operational systems maintained an enduring impact, which reached a 1.71 odds ratio, while digital policies, leadership frameworks and competence development strategies affected educational results through system-wide changes. The low heterogeneity indicates that institutional mechanisms function throughout different situations in a more standardised manner than individual psychological factors. The success of digital transformation projects depends on educational governance systems, which operate at a macro level.

The research method requires cross-sectional studies and SEM-based designs because they prevent researchers from establishing causal relationships between factors, which self-reported data creates measurement challenges. The research included experimental studies, longitudinal studies, and learning analytics studies, but these studies remain insufficiently represented. The different population types that researchers selected for their sampling methods created separate research domains. The study results demonstrate that publication bias does not exist in most research groups, which enhances the overall study outcomes. Digital transformation in education enables educational institutions to achieve psychological and institutional development, which especially benefits student engagement and cognitive learning processes. The effect of digital transformation on motivation and self-regulation depends on specific contextual factors. The research results show that digital transformation success depends on three factors, which include the teaching system design, student attributes, and institutional help resources. The research team should study three specific areas, which include longitudinal research, cross-cultural research, and behavioural analytics research, because these areas will help them understand how digital environments influence psychological development.

### Comparison with previous meta-analyses

The association between digital transformation and enhancements in educational psychology has remained constant, and the current findings have largely been found to be in agreement with earlier systematic reviews and meta-analyses. Nevertheless, whereas the earlier reviews only focused on one technology or one aspect of educational psychology, the current research has focused on five interlinked aspects.

Learning engagement proved to be the most effective predictor (OR = 1.79), which is consistent with earlier studies confirming that technology-assisted learning contributes to the increased participation of students and their success. According to Doo and Kim (2024), there exists a strong positive correlation between the level of learning engagement and learning results in e-learning environments at the college level, whereas Gosain et al. (2023) have shown how interactive e-teaching techniques positively influence the participation of students. The use of AI-assisted gamified learning environments to increase personalization and engagement of students has been confirmed by Niyazova et al. (2026).

Furthermore, our results regarding motivation and affective psychology (OR = 1.57) were in accordance with previous studies yet were characterized by high heterogeneity, meaning that motivational impacts depend on the contextual characteristics. This finding is in line with the findings of Niyazova et al. (2026) and Cho et al. (2022) stating that instructional design, institutional support, and efficient digital pedagogy have great influence on the motivational impact of educational games.

Positive influence on cognitive and metacognitive impacts (OR = 1.79) was confirmed by the meta-analysis conducted by Barz et al. (2024), proving that digital game-based learning contributes greatly to the improvement of higher order cognitive skills, and by the earlier review conducted by Clark et al. (2016), proving that educational games improve knowledge acquisition and problem-solving skills. Still, publication bias found by us in this regard requires caution in interpreting these results.

Our findings show that institutional variables (OR = 1.71) and self-regulation (OR = 1.66) have significant influence on successful digital transformation. These findings are consistent with Cho et al. (Cho et al., 2022) in that psychological sustainability cannot be achieved through technological use alone but must be supplemented by institutional policies, good instruction, and proper digital infrastructure. In general, our research builds on existing literature by providing an extensive review of various psychological perspectives and demonstrates that digital transformation is influenced by the interaction of educational technology, learner characteristics, and institutions.

### Strengths

- The meta-analysis examines 42 studies that investigate various psychological and institutional domains.
- The random-effects model with the inverse variance method establishes accurate study results for different research designs.
- The research study demonstrates its global evidence base, which increases its external validity and cross-cultural applicability.
- The research study examined five different domains, which showed both psychological and structural characteristics.
- The research study uses funnel plots together with Egger’s test to assess publication bias, which increases its methodological strength.
- The research study combines different research methodologies through SEM, experimental, longitudinal and analytics methods to create comprehensive research results.

### Limitations

- The research study depends on cross-sectional and SEM-based studies, which prevent researchers from making causal connections.
- The research study showed that people exhibit different degrees of motivation and self-regulation across various contexts.
- Cognitive and metacognitive psychology research exhibits publication bias according to evidence.
- Research studies that depend on participant self-reported information through surveys face an increased risk of measurement error.
- The research study faces temporal validity issues because it includes too few longitudinal and experimental research studies.
- The research studies used different sampling methods, which creates problems for comparing their results.
- As this was a single-author review, independent duplicate screening, data extraction, and risk-of-bias assessment were not available, which may have increased the possibility of reviewer error.

### Future prospects

- The research field needs longitudinal and experimental studies, which will help establish causal connections.
- Research teams should use learning analytics together with AI-driven behavioural data to objectively assess learning outcomes.
- The study aims to explore how digital transformation affects different cultures across various nations.
- The study aims to develop hybrid models that will combine psychological, technological, and institutional factors.
- The research study needs to create standardised measurement tools that will assess student engagement, motivation, and self-regulation.
- The educational sector needs to focus on digital well-being because it helps people learn to manage the advantages and disadvantages associated with educational technology.

## Conclusion

Through this meta-analysis, researchers demonstrate that educational institutions achieve their psychological and institutional objectives through digital transformation, which brings them positive results. The research showed that learning engagement and institutional factors produced the strongest effects because these elements maintained their most accurate results. The study found strong cognitive and metacognitive effects, but publication bias created a need for researchers to interpret the results with caution. Digital transformation functions as a major force for educational progress, yet its impact depends on three factors, which include the teaching methods used and the characteristics of students and educational institutions. Future educational strategies must implement three components, which include context-specific execution, digital design and evidence-backed technology implementation for teaching and learning purposes.

## Data Availability

The raw data supporting the conclusions of this article will be made available by the authors, without undue reservation.
